# Leveraging Marine Predators Algorithm with Deep Learning for Lung and Colon Cancer Diagnosis

**DOI:** 10.3390/cancers15051591

**Published:** 2023-03-03

**Authors:** Hanan Abdullah Mengash, Mohammad Alamgeer, Mashael Maashi, Mahmoud Othman, Manar Ahmed Hamza, Sara Saadeldeen Ibrahim, Abu Sarwar Zamani, Ishfaq Yaseen

**Affiliations:** 1 Department of Information Systems, College of Computer and Information Sciences, Princess Nourah bint Abdulrahman University, P.O. Box 84428, Riyadh 11671, Saudi Arabia; 2 Department of Information Systems, College of Science & Art at Mahayil, King Khalid University, P.O. Box 960, Muhayil 63311, Saudi Arabia; 3 Department of Software Engineering, College of Computer and Information Science, King Saud University, P.O. Box 103786, Riyadh 11543, Saudi Arabia; 4 Department of Computer Science, Faculty of Computers and Information Technology, Future University in Egypt, New Cairo 11835, Egypt; 5 Department of Computer and Self Development, Preparatory Year Deanship, Prince Sattam Bin Abdulaziz University, P.O. Box 173, Al-Kharj 16242, Saudi Arabia

**Keywords:** lung cancer, colon cancer, computer-aided diagnosis, MobileNet, marine predator’s algorithm

## Abstract

**Simple Summary:**

The histopathological detection of these malignancies is a vital element in determining the optimal solution. Timely and initial diagnosis of the sickness on either front diminishes the possibility of death. Deep learning (DL) and machine learning (ML) methods are used to hasten such cancer recognition, allowing the research community to examine more patients in a much shorter period and at a less cost.

**Abstract:**

Cancer is a deadly disease caused by various biochemical abnormalities and genetic diseases. Colon and lung cancer have developed as two major causes of disability and death in human beings. The histopathological detection of these malignancies is a vital element in determining the optimal solution. Timely and initial diagnosis of the sickness on either front diminishes the possibility of death. Deep learning (DL) and machine learning (ML) methods are used to hasten such cancer recognition, allowing the research community to examine more patients in a much shorter period and at a less cost. This study introduces a marine predator’s algorithm with deep learning as a lung and colon cancer classification (MPADL-LC3) technique. The presented MPADL-LC3 technique aims to properly discriminate different types of lung and colon cancer on histopathological images. To accomplish this, the MPADL-LC3 technique employs CLAHE-based contrast enhancement as a pre-processing step. In addition, the MPADL-LC3 technique applies MobileNet to derive feature vector generation. Meanwhile, the MPADL-LC3 technique employs MPA as a hyperparameter optimizer. Furthermore, deep belief networks (DBN) can be applied for lung and color classification. The simulation values of the MPADL-LC3 technique were examined on benchmark datasets. The comparison study highlighted the enhanced outcomes of the MPADL-LC3 system in terms of different measures.

## 1. Introduction

Cancer is a common disease where abnormal cells start to develop in an uncontrolled manner, which starts in any tissue or organ of the body. Cancer ranks as the second leading factor of death worldwide, accounting for nearly 9.6 million deaths in 2018 [1,2,3]. Among several cancer types, lung cancer denotes 1.76 million passings and 2.06 million cases, whereas colorectal malignancy accounts for 783 thousand deaths and 1.80 million cases. Non-small-cell cancer (NSCLC) and small-cell cancer in the lungs (SCLC) are the two kinds of lung cancer [4,5] which abruptly spread and develop. SCLC remains a dangerous form of cancer from cells displaying neuroendocrine qualities and is recorded for fifteen percent of total lung cancer cases. NSCL accounts for 85% of total cases and is further divided into 3 pathology types; they are enormous cell carcinoma, adenocarcinoma, and squamous cell carcinoma [6]. So, accurate and timely diagnosis of lung cancer histology was an urgent need since its treatment relies upon the histology types, stage of the disease, and molecular profile; it was found to be vital for analyzing the histopathology imageries of lung cancer. Yet, manual analysis of histopathology reports is subjective and time-taking [7,8,9,10].

Nowadays, the technical advancement in the domain of medical image and image processing has presented a lot of effective and cost-effective computer-aided diagnostics methods [11,12]. The end goal of the old technique was to execute a pattern-recognition-related mechanism for automatic cancer diagnosis. The technique extracts a standard set of handcrafted features from histology imageries and trained classifiers’ over-extracted features for categorizing the tumorous cells [13]. These days, medical image processing has grabbed the attention of many in deep neural networks (DNNs), which combines classification and feature extraction within a unified learning structure [14,15,16]. DNN has successfully shown great tasks in image segmentation, image classification, and object recognition. Convolutional neural networks (CNNs), which were DNN approaches, were broadly utilized in computer vision (CV) tasks because of their auspicious success in target recognition and classification [17]. The performance is based on the depth of CNN. However, increasing the CNN depth can cause problems with saturated accuracy and vanishing gradient, which becomes a network challenge. DNNs have positively shown great achievements in image segmentation, image classification, and object recognition [18,19,20].

This study introduces a marine predator’s algorithm with deep learning as a lung and colon cancer classification (MPADL-LC3) technique. The presented MPADL-LC3 technique employs CLAHE-based contrast enhancement as a pre-processing step. In addition, the MPADL-LC3 technique applies MobileNet to derive feature vector generation. Meanwhile, the MPADL-LC3 technique employs MPA as a hyperparameter optimizer. Furthermore, deep belief networks (DBN) can be applied for lung and color classification. The simulation values of the MPADL-LC3 technique are examined on benchmark datasets in terms of different measures.

The rest of the paper is given as follows. Section 2 provides a detailed literature review, and Section 3 offers the proposed model. Then, Section 4 elaborates on the performance validation, and Section 5 concludes the work.

## 2. Related Works

This section offered a detailed literature review of existing lung and colon detection techniques. A new optimized hybrid DL and ML architecture is developed in [21]. This architecture comprises two stages. At first, the features of lung and colon histopathological images (HSI) were mined by the PCA network. Next, classification was performed by using the ELM algorithm with the ROA that categorizes lung cancer and CC into five different types. Hoang et al. [22] developed a modified DNN transfer learning for lung cancer, and CC classification relies upon GoogLeNet. Particularly, the fundamental concept of the Inception model of GoogLeNet runs convolution and pooling operations with different filter sizes simultaneously such that there is no need to face any trade-offs. The next advantage of the Inception model is overparameterization dealing and dimensionality reduction of feature maps.

Attallah et al. [23] developed an architecture based on a lightweight DL approach for the earlier detection of lung cancer and CC. The architecture uses different transformation techniques that implement feature reduction and offer a broader representation of the data. In that regard, HSI is fed into the SqueezeNet, ShuffleNet, and MobileNet methods. The amount of deep features attained from the model is consequently decreased using fast Walsh–Hadamard transform (FHWT) and PCA models. Next, the DWT model is used for fusing the FWHT-reduced features attained from three different DL algorithms. In Toğaçar [24], AI-supported models and optimization techniques have been used for realizing the classification of lung cancer and CC HSI. In this work, the image class was trained from scratch with DarkNet-19, which is one of the DL techniques.

Mangal et al. [25] presented a computer-assisted diagnosis technique for detecting squamous cell carcinomas and adenocarcinomas of the lung and colon using the CNN network by estimating the digital pathology image for cancer. A shallow NN was used for classifying HSI into squamous cell carcinomas, benign, and adenocarcinomas for the lung. Mehmood et al. [26] developed a computationally efficient and highly accurate method for swift and precise detection of lung cancer and CC as a substitute for the cancer detection method. A massive dataset of lung and colon HSI was used for the validation and training process [27]. A CNN-based method was developed for the classification of lung cancer and CC image datasets utilizing two common optimizer techniques: RMSprop and Adam. In this work, a separate model was constructed for lung cancer and CC through CNN for more accurately predicting the types of the disease.

## 3. The Proposed Model

**Figure 1:**
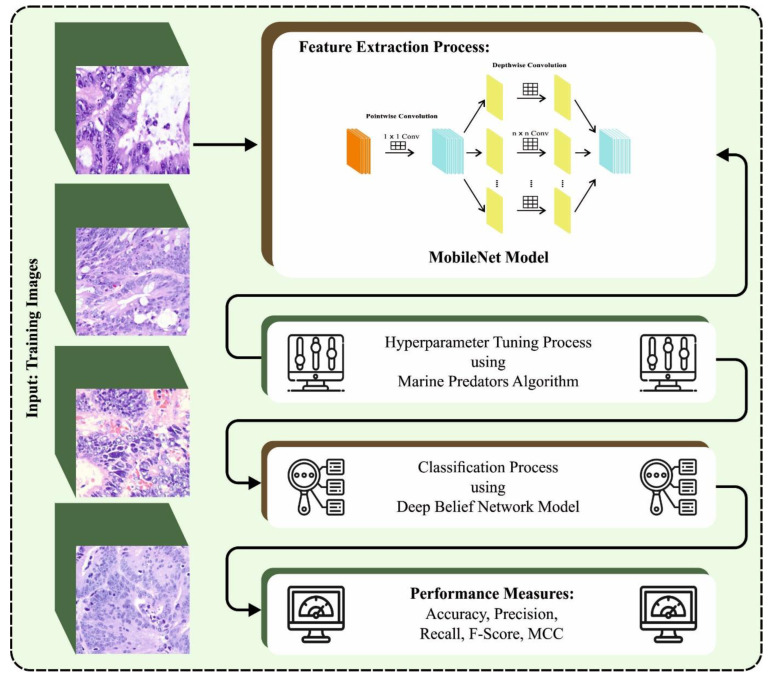
The overall flow of the MPADL-LC3 approach.

In this study, we have introduced a new MPADL-LC3 algorithm for lung and colon cancer classification. The presented MPADL-LC3 method aims to properly discriminate different types of lung and colon cancer in histopathological images. To accomplish this, the MPADL-LC3 technique encompasses CLAHE-based contrast enhancement, MobileNet feature extraction, MPA-based hyperparameter tuning, and DBN-based classification. Figure 1 illustrates the overall flow of the MPADL-LC3 approach.

### 3.1. Contrast Enhancement

Primarily, the contrast enhancement uses the CLAHE technique. CLAHE has been primarily employed for enriching low-contrast medical imageries [28]. CLAHE varies from normal AHE in that it limits contrast. To address the problem of noise amplification, the CLAHE enforced clipping limits. Before computation of the Cumulative Distribution Function, the CLAHE limits the intensification by clipping the histogram at a predefined value (CDF). The CLAHE approach has divided input original images into non-overlapping contextual areas called sub-images, blocks, or tiles. The CLAHE can be described by two variables: Clip Limit (CL) and Block Size (BS). These two variables chiefly govern enhanced image quality. If CL is amplified, the image becomes brighter as the input images contain a very low intensity, and larger CL makes its histogram flatter.

### 3.2. Feature Extraction Using Optimal MobileNet

In this study, the MobileNet model is employed for feature vector generation. CNN is an effective network type of DNN to deal with a considerable quantity of difficulty around the computation and pre-processing of data [29]. The major component of the CNN entails dropout, convolutional, pooling, flattening, and nonlinear activation layers. The convolutional layer mines the feature map out of input images, which are the main layer in CNN. The flattening layer transforms (flattens) the dataset into an array; thus, the dense layer performs data computation. The pooling layer, termed a sub-sampling layer, is a major component of CNN. The pooling layer acts on the feature map extracted through the convolution layer. It decreases the feature size for extracting the relevant feature from the feature map to prevent overfitting. Pooling can be a sum, max, or average. The max-pooling can find more sharp features than the sum and average pooling.

MobileNet is a lightweight DNN structure with higher classification accuracy and fewer parameters. It is a CNN architecture for mobile vision applications and image classification. MobileNet applies depthwise separable convolution in every color channel instead of merging all three and flattening them. The depthwise separable layer is divided into two layers, a separate layer of filtering and a separate layer for compiling. This factorization of the MobileNet model decreases the model and computational size. MobileNet is better suited for embedded systems since it needs lower computational power to run and is efficient in the healthcare field. This study develops artificially intelligent medical devices based on MobileNet architecture that takes lower computational power with optimum time and provides higher accuracy. The MobileNet model is suitable for embedded vision applications. An additional feature of the MobileNet model is two global hyperparameters that effectively present the trade-offs between latency and accuracy.

For an optimal hyperparameter tuning process, the MPA is involved in this study. The MPA is a population, iterative-based optimization technique [30]. At first, an initial population of the solution is generated. The population matrix of n×d size can be produced by:

(1) $$P=\left[\begin{array}{cccc} X_{1,1} & X_{1,2} & \ldots & X_{1,d}\\ X_{2,1} & X_{2,1} & \ldots & X_{2,d}\\ \vdots & \vdots & \vdots & \vdots \\ X_{n,1} & X_{n,2} & \ldots & X_{n,d} \end{array}\right]$$

In Equation (1), n refers to the population size, viz, the number of searching agents (every prey and predator are looking for food and regarded as a searching agent), and d denotes the dimension (number of parameters) of every agent. Every parameter of the initial solution was distributed uniformly over searching space as follows:

(2) $$X_{j}=l_{b}+rand\times \left(u_{b}-l_{b}\right),$$

In Equation (2), $l_{b}$ and $u_{b}$ represent the lower and upper bounds, and rand indicates a uniform distribution random integer. The topmost predator has the better foraging abilities according to the concept of survival of the fittest. Thus, the best solution was selected as a better predator and utilized for constructing a matrix named Elite.

(3) $$Elite=\left[\begin{array}{llll} X_{1,1}^{I} & X_{1_{,}2}^{I} & \ldots & X_{1,d}^{I}\\ X_{2_{,}1}^{I} & X_{2,2}^{I} & \ldots & X_{2_{,}d}^{I}\\ \vdots & \vdots & \vdots & \vdots \\ X_{n_{,}1}^{I} & X_{n_{,}2}^{I} & \ldots & X_{n_{,}d}^{I} \end{array}\right]=\left[\begin{array}{l} \bar{X}\\ \bar{X}\\ \vdots \\ \bar{X} \end{array}\right],$$

In Equation (3), $\begin{array}{c} \bar{X} \end{array}$ denotes the topmost predator vector that is repeated n times to create an Elite matrix. The Elite matrix would be upgraded at the end of every iteration if the optimal predator of the population was swapped by the best predator. Another matrix called Prey was produced by a similar dimension as the Elite.

(4) $$Prey=\left[\begin{array}{llll} X_{1,1} & X_{1_{,}2} & \ldots & X_{1,d}\\ X_{2_{,}1} & X_{2,2} & \ldots & X_{2_{,}d}\\ \vdots & \vdots & \vdots & \vdots \\ X_{n_{,}1} & X_{n_{,}2} & \ldots & X_{n_{,}d} \end{array}\right],$$

where $X_{i,j}$ represents the jth dimension of $i^{t}$s prey. During the first iteration, the Prey matrix is equal to the randomly generated population matrix P. In all subsequent iterations, the Prey is updated, and its values are used to compute the Elite matrix. The update of the Prey matrix is carried out separately in three phases of MPA optimization.

Phase 1: This stage agrees to a higher velocity ratio and occurs at the first $\left(\frac{1}{3}\right)th$ of maximal iteration where exploration is greater. The updating rule can be represented as:

(5) $$\bar{Stepsize_{i}}=\bar{R_{B}}⊗\left(\bar{Elite_{i}}-\bar{R_{B}}⊗\bar{Prey_{i}}\right),\forall i=1,\cdots ,n$$

(6) $$\bar{Prey_{i}}=\bar{Prey_{i}}+P.R⊗\bar{Stepsize_{i}},\forall i=1,\cdots ,n$$

From the expression, $\bar{Prey_{i}}$ denotes the vector of the Prey matrix, and $\bar{R_{B}}$ and R show the vector of d dimension comprising arbitrary numbers from Normal and Uniform distribution, correspondingly. P denotes a constant equivalent to 0.5, and ⊗ indicates component-wise multiplication.

Phase 2: This stage agrees to the unit velocity ratio once the prey and predator move at a similar pace and happens for the intermediate ${(}_{3}^{1})rd$ of the iteration, where exploration and exploitation matter. The updating rule can be represented as:

(7) $$\bar{Stepsize_{i}}=\bar{R_{L}}⊗\left(\bar{Elite_{i}}-\bar{R_{L}}⊗\bar{Prey_{i}}\right),\forall i=1,\cdots ,\frac{n}{2}$$

(8) $$\bar{Prey_{i}}=\bar{Prey_{i}}+P\bar{R}⊗\bar{Stepsize_{i}},\forall i=1\cdots ,\frac{n}{2}$$

(9) $$\bar{Stepsize_{i}}=\bar{R_{B}}⊗\left(\bar{Elite_{i}}-\bar{R_{B}}⊗\bar{Prey_{i}}\right),\forall i=\frac{n}{2}+1,\cdots ,n$$

(10) $$\bar{Prey_{i}}=\bar{Elite_{i}}+P.CP⊗\bar{Stepsize_{i}},\forall i=\frac{n}{2}+1\cdots ,n$$

Now, $\bar{R_{L}}$ indicates a vector of size d comprising a random number depending on Lévy distribution, $CF=(1-\frac{I}{I_{Max}}{)}^{\left(2\frac{l}{l_{Max}}\right)}$ denotes the adaptive parameter used for controlling the step size for predator movement, I indicates the existing iteration, and $I_{Max}$ represents the maximal amount of iterations.

Phase 3: This stage agrees to a lower velocity ratio once the predator moves faster when compared to the prey. This phase occurs at the last ${(}_{3}^{1})rd$ iteration where exploitation matters. The updating rule can be represented as:

(11) $$\bar{Stepsize_{i}}=\bar{R_{L}}⊗\left(\bar{Elite_{i}}-\bar{Prey_{i}}\right),\forall i=1,\cdots ,n$$

(12) $$\bar{Prey_{i}}=\bar{Elite_{i}}+P.CP⊗\bar{Stepsize_{i}},\forall i=1\cdots ,n$$

Next, the behavioral change in MPs is modeled due to environmental effects. This effect is called fish aggregating devices (FADs) and is also represented as local optimal; thus, the prey and predator should implement a long jump during simulation to prevent stagnation in local optimal. The updating rule of the Prey matrix can be mathematically expressed:

(13) $$\bar{Prey_{i}}=\{\bar{\frac{Prey_{i}}{Prey_{i}}}\frac{ax^{-\bar{X}}}{Prey_{r1}}\frac{n)]⊗}{Prey_{r2}}+\bar{R}⊗\bar{X_{mmi}}\bar{U}+((-)r>PADs)\}\;\bar{Prey_{i}}=\left\{\begin{array}{cc} \bar{Prey_{i}}+CF[\bar{X_{min}}+\bar{R}⊗\left(\bar{X_{max}}-\bar{X_{min}}\right)⊗\bar{U} & r\leq FADs\\ \bar{Prey_{i}}+\left[FAD(1-r)r\right]+\left(\bar{Prey_{r1}}-\bar{Prey_{r2}}\right) & r>FADs \end{array}\right\},$$

where FADs=0.2 denotes the existence probability of the FAD effect; U represents a randomly produced binary vector; r indicates the uniformly distributed random integer in [0,1]; ${and\;X}_{max}$ and $X_{min}$ indicate the vector has minimum and maximum boundaries of dimensions, correspondingly; and r1, and r2 denote random numbers of Prey matrix.

Afterward, the Prey matrix was upgraded based on Equations (6) to (12), and integrating the FAD effect of Equation (13), these matrices are assessed for fitness functions. The fitness of every solution of the present iteration was compared with its corresponding solutions at the previous iteration. When the present solution was better, they replaced the earlier one. At the following iteration, the better solution of Prey generates the Elite matrix and upgrades the Prey matrix based on Equations (6) to (12).

The MPA approach has derived fitness functions for obtaining enhanced classifier outcomes. It determined positive values for indicating the superior outcome of the candidate solutions. Here, the reduced classifier error rate was treated as the fitness function, as specified in Equation (14).

(14) $$fitness\left(x_{i}\right)=Classifier\;Error\;Rate\left(x_{i}\right)=\frac{number\;of\;misclassified\;samples}{Total\;number\;of\;samples}\;*\;100,$$

### 3.3. Classification Model Using DBN Model

**Figure 2:**
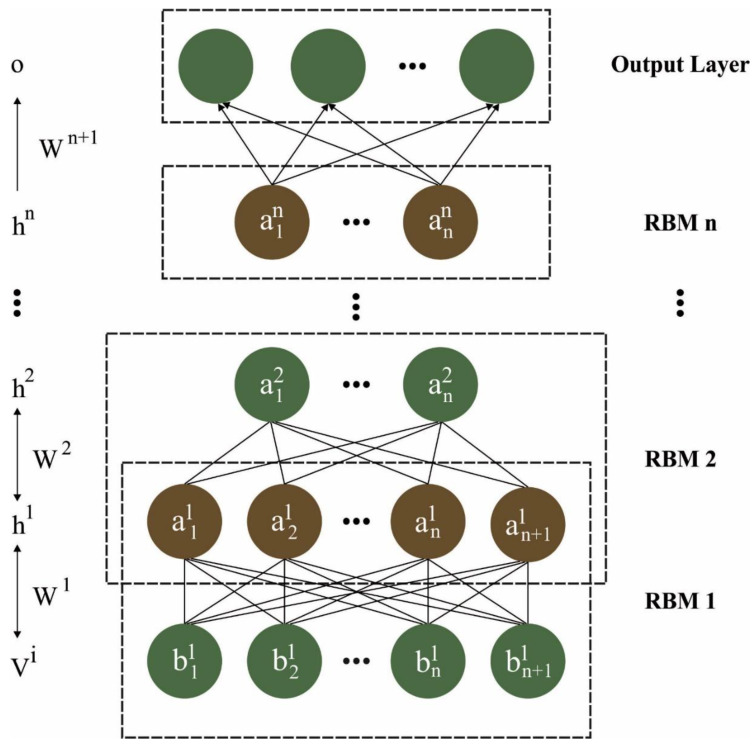
Structure of DBN.

In the final phase, the DBN method can be used for lung and color classification. DBN is a probabilistic generalization model collected by the stacked module of RBM and provides an alternative to the discriminatory nature of classical NN [31]. The most important feature of DBN is the capability to encode higher-request network structure and quick induction. The DBN model used two probabilities and unassisted solving to deliver output. It is made up of double inert factors, and they have coordinated and undirected layers. Different from other models, every layer in DBN learns the complete data. It is used for clustering, identification, image processing, signal-capture data, and video sequels in addition to training non-linear autoencoder (AE). Figure 2 illustrates the framework of DBN. The mathematical modeling of DBN is given in the following: A DBN with l hidden layer (HL) contains l weight matrices $W^{\left(1\right)},\ldots ,W^{\left(l\right)}$; also, it has l+1 bias vector $b^{\left(0\right)},\ldots ,b^{\left(1\right)},$ where $b^{\left(0\right)}$ provides the bias for the visible layer shown below:

(15) $$P\left(h^{\left(l\right)},h^{\left(l-1\right)}\right)\propto exp\left(b^{(l{)}^{T}}h^{\left(l\right)}+b^{(l-1{)}^{T}}h^{\left(l-1\right)}+h^{(l-1{)}^{T}}W^{\left(l\right)}h^{\left(l\right)}\right),$$

(16) $$\left(h_{i}^{\left(k\right)}=1|h^{\left(k+1\right)}\right)=\sigma \left(b_{i}^{\left(k\right)}+W_{:,i}^{(k+1{)}^{T}}h^{\left(k+1\right)}\right),$$

where ∀i,∀k∈1,…,l−2

(17) $$P\left(v_{i}=1|h^{\left(1\right)}\right)=\sigma \left(b_{i}^{\left(0\right)}+W_{:,i}^{(1{)}^{T}}h^{\left(1\right)}\right)\forall i,$$

In the case of the real-valued visible unit, replace

(18) $$v∼N\left(b^{\left(0\right)}+W^{(1{)}^{T}}h^{\left(l\right)}\beta^{-1}\right),$$

with β diagonal for tractability σ(x)=1/(1+exp(−x)). The weight from the trainable DBN is utilized as the initialized weight of the DNN:

(19) $$h^{\left(1\right)}=\sigma (b^{\left(1\right)}+v^{T}W^{\left(1\right)}),$$

(20) $$h^{\left(l\right)}=\sigma \left(b_{i}^{\left(l\right)}+h^{(l-1{)}^{T}}W^{\left(l\right)}\right),\forall l\in 2,\ldots ,m,$$

Additionally, the entire load is tweaked by using backpropagation or other discriminatory modules to improve the effectiveness of the algorithm.

An AE–NN can adaptively discover data characteristics and later characterize the complicated data in an effective manner that improves the accuracy and training speed [32]. Thus, the study presents an AE layer to mine features from $X^{\prime }$ pre-processed data. It has an encoder and decoder procedure. These two procedures are NN with a similar structure. The input and output layers have a similar number of nodes and similar meanings. The encoding layer reduces the number of dimensions of the input dataset $X^{\prime }$ to the HL, and then the decoding layer will decode the HL to $X^{\prime }$, whereby the error between $X^{\prime }$ and ${\tilde{X}}^{\prime }$ should be smaller. The encoder process can be mathematically expressed as follows:

(21) $$\begin{array}{l} E_{1}=f(W_{1}\times X^{\prime }+b_{1})\\ E_{2}=f(W_{2}\times E_{1}+b_{2})\\ \ldots \\ E_{n}=f(W_{n}\times E_{n-1}+b_{n}) \end{array},$$

Furthermore, the decoder process can be mathematically expressed as follows:

(22) $$\begin{array}{l} D_{1}=f(W_{1}^{\prime }\times E_{n}+b_{1}^{\prime })\\ \ldots \\ D_{n-1}=f(W_{n-1}^{\prime }\times D_{n-2}+b_{n-1}^{\prime })\\ D_{n}=f(W_{n}^{\prime }\times D_{n-1}+b_{n}^{\prime })\\ X^{\prime }=f(W_{n+1}^{\prime }\times D_{n}+b_{n+1}^{\prime }) \end{array},$$

where $\left(w_{1},w_{2},\ldots w_{n}\right)$ and $\left(b_{1},b_{2},\ldots b_{n}\right)$ represent the weight and bias in the encoding stage, $\left(w_{1}^{\prime },w_{2}^{\prime },\ldots ,w_{n}^{\prime }\right)$ and $\left(b_{1}^{\prime },b_{2}^{\prime },\ldots b_{n}^{\prime }\right)$ signify weights and biases in decoding stage, and n defines the count of encoding and decoding layers. The objective function is as given in Equation (23) to train the suitable parameter, whereas N denotes the number of input datasets for batch processing. Lastly, $E_{n}$ is utilized as an input to the GRU layer as follows:

(23) $$L\left(\tilde{X^{\prime }},X^{\prime }\right)=\sum \frac{(\tilde{X^{\prime }}-X^{\prime }{)}^{2}}{N},$$

## 4. Results and Discussion

### 4.1. Data Used

**Figure 3:**
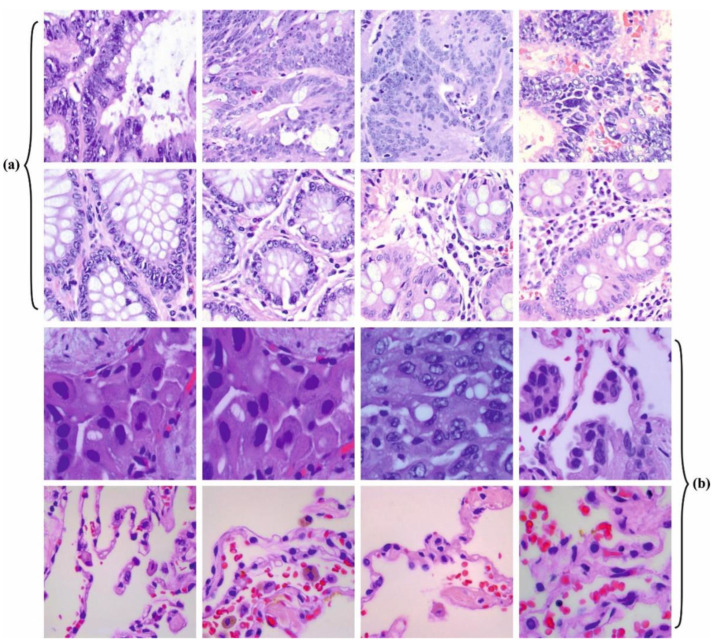
Sample Images: (**a**) Colon and (**b**) Lung.

In this section, the lung and colon cancer classification results of the MPADL-LC3 technique can be examined on a dataset comprising 25,000 HIs [33]. The details relevant to the dataset are reported in Table 1. Figure 3 represents the sample image of the colon and lung.

### 4.2. Result Analysis

**Figure 4:**
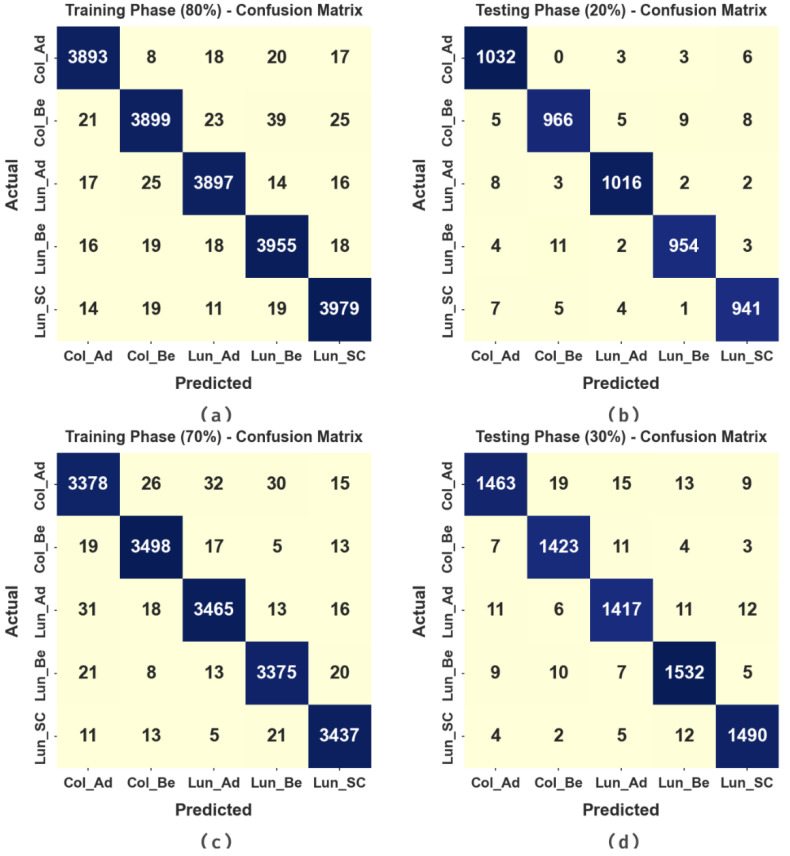
Confusion matrices of MPADL-LC3 approach: (**a**,**b**) TRS/TSS of 80:20 and (**c**,**d**) TRS/TSS of 70:30.

In Figure 4, the confusion matrices of the MPADL-LC3 technique on colon and lung cancer classification are reported. The results indicate that the MPADL-LC3 technique has accurately identified lung and colon cancer types.

**Figure 5:**
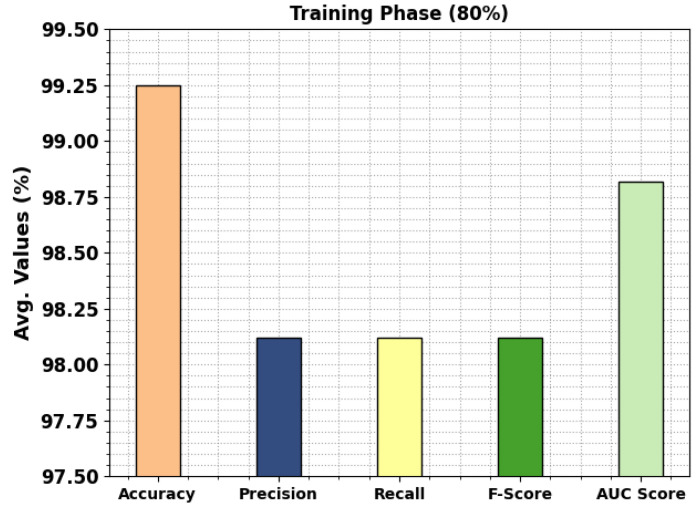
Average outcomes of MPADL-LC3 algorithm on 80% of TRS.

In Table 2, the overall colon and lung cancer classification outcomes of the MPADL-LC3 technique with 80:20 of TRS/TSS are offered. In Figure 5, the classification results of the MPADL-LC3 method on 80% of TRS are provided. The results represented that the MPADL-LC3 technique has provided effectual outcomes under all classes. It is highlighted that the MPADL-LC3 technique reaches an average $accu_{y}$ of 99.25%, $prec_{n}$ of 98.12%, $reca_{l}$ of 98.12%, $F_{score}$ of 98.12%, and $AUC_{score}$ of 98.82%.

**Figure 6:**
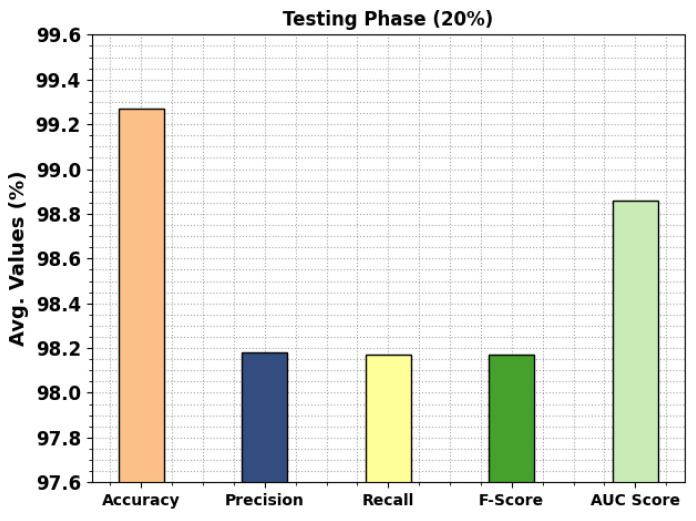
Average outcomes of MPADL-LC3 algorithm on 20% of TSS.

In Figure 6, the classification outcomes of the MPADL-LC3 approach on 20% of TRS are provided. The outcomes designated in the MPADL-LC3 system have rendered effectual outcomes under all classes. It is pointed out that the MPADL-LC3 method reaches an average $accu_{y}$ of 99.27%, $prec_{n}$ of 98.18%, $reca_{l}$ of 98.17%, $F_{score}$ of 98.17%, and $AUC_{score}$ of 98.86%.

**Figure 7:**
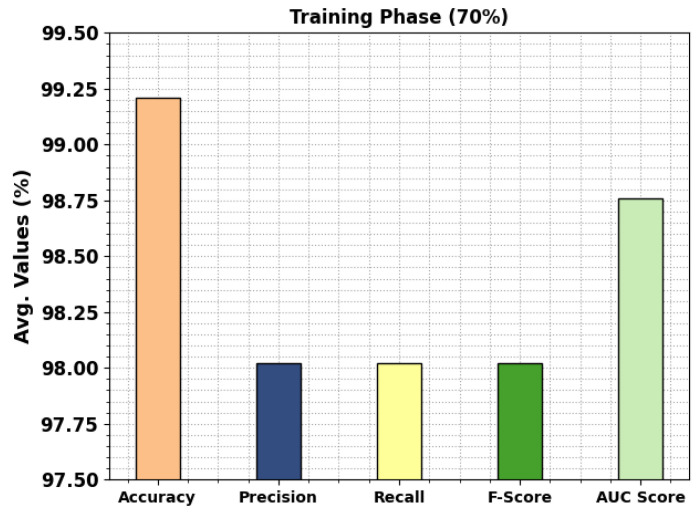
Average outcomes of MPADL-LC3 algorithm on 70% of TRS.

In Table 3, the overall colon and lung cancer classification outcomes of the MPADL-LC3 technique with 70:30 of TRS/TSS are provided. In Figure 7, the classification results of the MPADL-LC3 method on 70% of TRS are offered. The results represented that the MPADL-LC3 technique has provided effectual outcomes under all classes. It is noted that the MPADL-LC3 algorithm attains an average $accu_{y}$ of 99.21%, $prec_{n}$ of 98.02%, $reca_{l}$ of 98.02%, $F_{score}$ of 98.02%, and $AUC_{score}$ of 98.76%.

**Figure 8:**
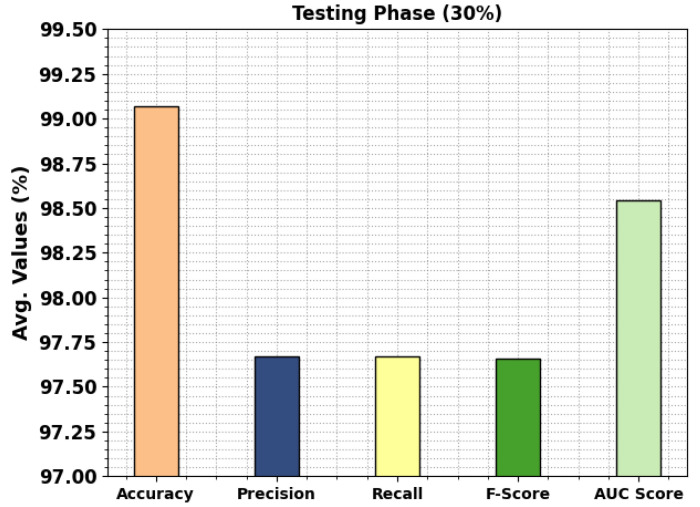
Average outcomes of MPADL-LC3 algorithm on 30% of TSS.

In Figure 8, the classification results of the MPADL-LC3 technique on 30% of TRS are provided. The outcomes signified that the MPADL-LC3 technique presented effectual outcomes under all classes. It is emphasized that the MPADL-LC3 approach reaches an average $accu_{y}$ of 99.07%, $prec_{n}$ of 97.67%, $reca_{l}$ of 97.67%, $F_{score}$ of 97.66%, and $AUC_{score}$ of 98.54%.

**Figure 9:**
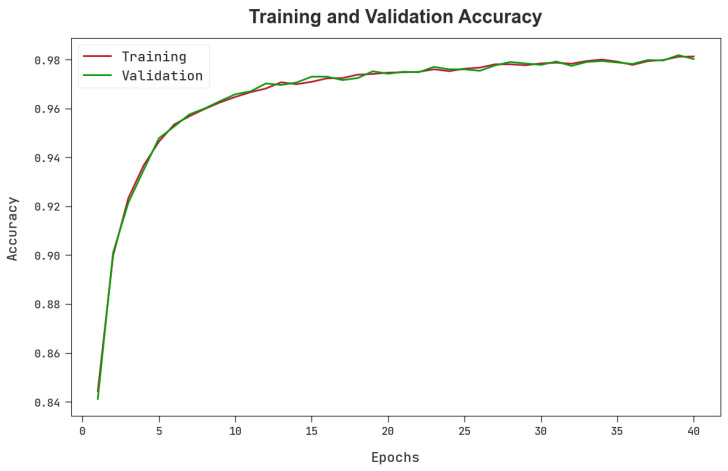
TACY and VACY outcomes of the MPADL-LC3 algorithm.

The TACY and VACY of the MPADL-LC3 approach are inspected on colon and lung cancer classification performance in Figure 9. The figure signified that the MPADL-LC3 method had improved performance with increased values of TACY and VACY. Visibly, the MPADL-LC3 model attained higher TACY outcomes.

**Figure 10:**
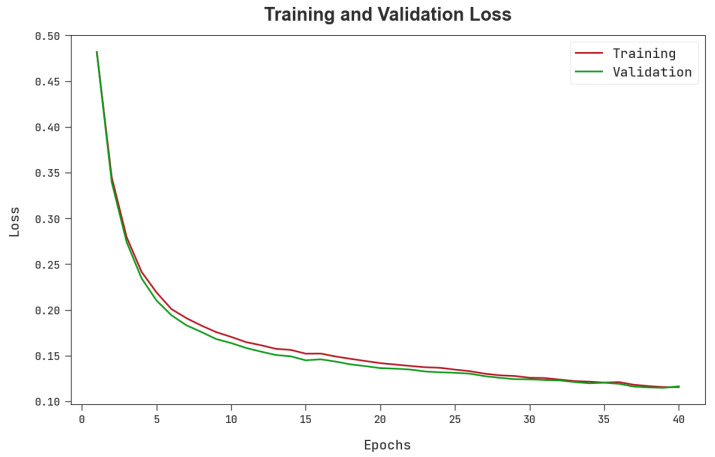
TLOS and VLOS outcomes of the MPADL-LC3 algorithm.

The TLOS and VLOS of the MPADL-LC3 technique are tested on colon and lung cancer classification performance in Figure 10. The figure implied that the MPADL-LC3 model exposed superior performance with minimum values of TLOS and VLOS. Particularly, the MPADL-LC3 approach has the fewest VLOS outcomes.

**Figure 11:**
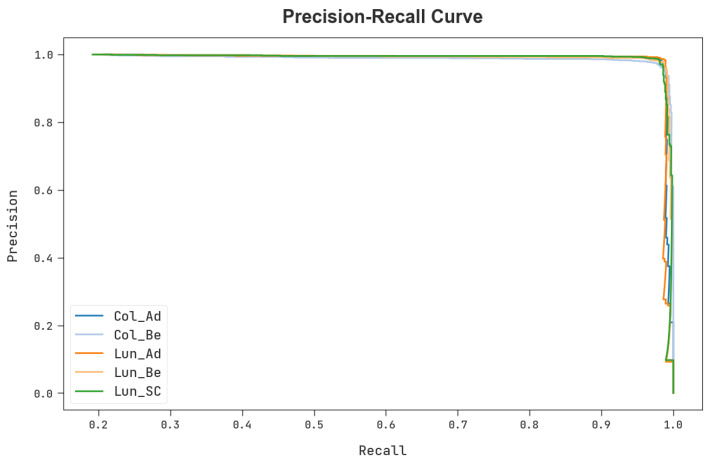
Precision–recall outcomes of the MPADL-LC3 algorithm.

A brief precision–recall investigation of the MPADL-LC3 system under the test database is shown in Figure 11. The results specified the MPADL-LC3 algorithm has improved values of precision–recall values under every class label.

### 4.3. Discussion

In Table 4, a comparison study of the MPADL-LC3 technique with recent DL models is carried out. The experimental results indicate that the mSRC model reports the fewest classifier outcomes. Meanwhile, the ResNet-50 model attains slightly improved outcomes, whereas the CNN and DL models report closer performance. Although the Faster RCNN and DAELGNN models accomplish closer performance with classification $accu_{y}$ of 98.64% and 98.73%, the MPADL-LC3 technique results in maximum outcomes with $accu_{y}$ of 99.27%. These results ensured the betterment of the MPADL-LC3 technique over other current techniques. The enhanced performance of the proposed model is due to the inclusion of the MPA-based hyperparameter tuning process.

## 5. Conclusions

In this study, we have introduced a new MPADL-LC3 approach for lung and colon cancer classification. The presented MPADL-LC3 algorithm aims to properly discriminate different types of lung and colon cancer in histopathological images. To accomplish this, the MPADL-LC3 technique employs CLAHE-based contrast enhancement as a pre-processing step. In addition, the MPADL-LC3 technique applied the MobileNet to derive feature vector generation. Meanwhile, the MPADL-LC3 technique introduced the MPA as a hyperparameter optimizer. Moreover, the DBN method is applied for lung and color classification. The simulation values of the MPADL-LC3 technique are examined on the benchmark dataset. The comparison study highlighted the enhanced outcomes of the MPADL-LC3 method with maximum accuracy of 99.27%. In the future, we plan to work on the architecture of the classification model and engineer new sets of features from more histopathological images to elevate its performance.

## Figures and Tables

**Table 1:** Details of the dataset.

Class Name	Description	No. of Samples
Col_Ad	Colon Adenocarcinoma	5000
Col_Be	Colon Benign Tissue	5000
Lun_Ad	Lung Adenocarcinoma	5000
Lun_Be	Lung Benign Tissue	5000
Lun_SC	Lung Squamous Cell Carcinoma	5000
**Total Number of Samples**		**25,000**

**Table 2:** Classifier outcomes of MPADL-LC3 approach on TRS/TSS of 80:20.

Labels	$Accu_{y}$	$Prec_{n}$	$Reca_{l}$	$F_{score}$	$AUC_{score}$
**Training Phase (80%)**					
Col_Ad	99.35	98.28	98.41	98.35	98.99
Col_Be	99.10	98.21	97.30	97.76	98.43
Lun_Ad	99.29	98.24	98.19	98.21	98.87
Lun_Be	99.19	97.73	98.24	97.98	98.83
Lun_SC	99.30	98.13	98.44	98.28	98.98
**Average**	**99.25**	**98.12**	**98.12**	**98.12**	**98.82**
**Testing Phase (20%)**					
Col_Ad	99.28	97.73	98.85	98.29	99.12
Col_Be	99.08	98.07	97.28	97.67	98.40
Lun_Ad	99.42	98.64	98.55	98.59	99.10
Lun_Be	99.30	98.45	97.95	98.20	98.79
Lun_SC	99.28	98.02	98.23	98.12	98.88
**Average**	**99.27**	**98.18**	**98.17**	**98.17**	**98.86**

**Table 3:** Classifier outcomes of MPADL-LC3 approach on TRS/TSS of 70:30.

Labels	$Accu_{y}$	$Prec_{n}$	$Reca_{l}$	$F_{score}$	$AUC_{score}$
**Training Phase (70%)**					
Col_Ad	98.94	97.63	97.04	97.33	98.23
Col_Be	99.32	98.18	98.48	98.33	99.01
Lun_Ad	99.17	98.10	97.80	97.95	98.66
Lun_Be	99.25	98.00	98.20	98.10	98.85
Lun_SC	99.35	98.17	98.57	98.37	99.05
**Average**	**99.21**	**98.02**	**98.02**	**98.02**	**98.76**
**Testing Phase (30%)**					
Col_Ad	98.84	97.93	96.31	97.11	97.90
Col_Be	99.17	97.47	98.27	97.87	98.83
Lun_Ad	98.96	97.39	97.25	97.32	98.31
Lun_Be	99.05	97.46	98.02	97.74	98.67
Lun_SC	99.31	98.09	98.48	98.28	99.00
**Average**	**99.07**	**97.67**	**97.67**	**97.66**	**98.54**

**Table 4:** Comparative outcome of MPADL-LC3 system with recent DL methods.

Methods	Accuracy	Precision	Recall	F-Score
MPADL-LC3	99.27	98.18	98.17	98.17
mSRC	88.31	85.14	91.66	86.70
Faster R-CNN	98.64	96.52	97.75	97.19
DAELGNN	98.73	97.98	96.47	96.65
RESNET-50	93.81	96.20	97.56	96.90
CNN	97.13	97.02	97.36	97.79
DL Model	96.34	96.94	96.31	98.03

## Data Availability

Data sharing does not apply to this article as no datasets were generated during the current study.

## References

[1] Talukder M.A., Islam M.M., Uddin M.A., Akhter A., Hasan K.F., Moni M.A. (2022). Machine learning-based lung and colon cancer detection using deep feature extraction and ensemble learning. Expert Syst. Appl..

[2] Veeresha P., Ilhan E., Prakasha D.G., Baskonus H.M., Gao W. (2021). Regarding on the fractional mathematical model of Tumour invasion and metastasis. Comput. Model. Eng. Sci..

[3] Hage Chehade A., Abdallah N., Marion J.M., Oueidat M., Chauvet P. (2022). Lung and colon cancer classification using medical imaging: A feature engineering approach. Phys. Eng. Sci. Med..

[4] Dubey R.S., Goswami P., Baskonus H.M., Gomati A.T. (2022). On the existence and uniqueness analysis of fractional blood glucose-insulin minimal model. Int. J. Model. Simul. Sci. Comput..

[5] Hasan I., Ali S., Rahman H., Islam K. (2022). Automated Detection and Characterization of Colon Cancer with Deep Convolutional Neural Networks. J. Healthc. Eng..

[6] Bawankar B.U., Chinnaiah K. (2022). Implementation of Ensemble Method on DNA Data Using Various cross Validation Techniques. BMC Bioinform..

[7] Pacal I., Karaboga D., Basturk A., Akay B., Nalbantoglu U. (2020). A comprehensive review of deep learning in colon cancer. Comput. Biol. Med..

[8] Gao W., Baskonus H.M. (2022). Deeper investigation of modified epidemiological computer virus model containing the Caputo operator. Chaos Solitons Fractals.

[9] Masud M., Sikder N., Nahid A.A., Bairagi A.K., AlZain M.A. (2021). A machine learning approach to diagnosing lung and colon cancer using a deep learning-based classification framework. Sensors.

[10] Trejos D.Y., Valverde J.C., Venturino E. (2022). Dynamics of infectious diseases: A review of the main biological aspects and their mathematical translation. Appl. Math. Nonlinear Sci..

[11] Jia X., Xing X., Yuan Y., Xing L., Meng M.Q.H. (2019). Wireless capsule endoscopy: A new tool for cancer screening in the colon with deep-learning-based polyp recognition. Proc. IEEE.

[12] Sabir Z., Umar M., Raja M.A.Z., Fathurrochman I., Hasan H. (2022). Design of Morlet wavelet neural network to solve the non-linear influenza disease system. Appl. Math. Nonlinear Sci..

[13] Ali M., Ali R. (2021). Multi-input dual-stream capsule network for improved lung and colon cancer classification. Diagnostics.

[14] Chen Q. (2022). Research on identifying psychological health problems of college students by logistic regression model based on data mining. Appl. Math. Nonlinear Sci..

[15] Al-Barzinji S.M. (2018). Diagnosis Lung Cancer Disease Using Machine Learning Techniques. Iraqi J. Inf. Technol. V.

[16] Ghufran M., Khan H.A., Ullah M., Ghufran S., Ayaz M., Siddiq M., Hassan S.S.U., Bungau S. (2022). In Silico Strategies for Designing of Peptide Inhibitors of Oncogenic K-Ras G12V Mutant: Inhibiting Cancer Growth and Proliferation. Cancers.

[17] Jiao Y., Li J., Qian C., Fei S. (2021). Deep learning-based tumor microenvironment analysis in colon adenocarcinoma histopathological whole-slide images. Comput. Methods Programs Biomed..

[18] Sharma A., Sharma L., Nandy S.K., Payal N., Yadav S., Vargas-De-La-Cruz C., Anwer M.K., Khan H., Behl T., Bungau S.G. (2023). Molecular Aspects and Therapeutic Implications of Herbal Compounds Targeting Different Types of Cancer. Molecules.

[19] Jiang D., Liao J., Duan H., Wu Q., Owen G., Shu C., Chen L., He Y., Wu Z., He D. (2020). A machine learning-based prognostic predictor for stage III colon cancer. Sci. Rep..

[20] Rahman M.M., Behl T., Islam M.R., Alam M.N., Islam M.M., Albarrati A., Albratty M., Meraya A.M., Bungau S.G. (2022). Emerging management approach for the adverse events of immunotherapy of cancer. Molecules.

[21] Naga Raju M.S., Srinivasa Rao B. (2023). Lung and colon cancer classification using hybrid principle component analysis network-extreme learning machine. Concurr. Comput. Pract. Exp..

[22] Hoang T.H., Binh N.T., Van V., Tan N.Q. (2022). Lung and Colon Tumor Classification Based on Transfer Learning-Based Techniques. Proceedings of the International Conference on Future Data and Security Engineering.

[23] Attallah O., Aslan M.F., Sabanci K. (2022). A framework for lung and colon cancer diagnosis via lightweight deep learning models and transformation methods. Diagnostics.

[24] Toğaçar M. (2021). Disease type detection in lung and colon cancer images using the complement approach of inefficient sets. Comput. Biol. Med..

[25] Mangal S., Chaurasia A., Khajanchi A. (2020). Convolution neural networks for diagnosing colon and lung cancer histopathological images. arXiv.

[26] Mehmood S., Ghazal T.M., Khan M.A., Zubair M., Naseem M.T., Faiz T., Ahmad M. (2022). Malignancy Detection in Lung and Colon Histopathology Images Using Transfer Learning with Class Selective Image Processing. IEEE Access.

[27] Zafar A., Nadeem M. (2022). Performance Evaluation of 2D CNN Optimizers for Lung and Colon Cancer Image Classification. Proceedings of the International Conference on Communication and Artificial Intelligence.

[28] Sahu S., Singh A.K., Ghrera S.P., Elhoseny M. (2019). An approach for de-noising and contrast enhancement of retinal fundus image using CLAHE. Opt. Laser Technol..

[29] Kumar A., Sharma A., Bharti V., Singh A.K., Singh S.K., Saxena S. (2021). MobiHisNet: A lightweight CNN in mobile edge computing for histopathological image classification. IEEE Internet Things J..

[30] Ahmad R., Awais M., Kausar N., Akram T. (2023). White Blood Cells Classification Using Entropy-Controlled Deep Features Optimization. Diagnostics.

[31] Nandakumar P., Narayan S. (2022). Cardiac disease detection using cuckoo search enabled deep belief network. Intell. Syst. Appl..

[32] Lian J., Dong P., Zhang Y., Pan J. (2020). A novel deep learning approach for tropical cyclone track prediction based on auto-encoder and gated recurrent unit networks. Appl. Sci..

[33] Borkowski A.A., Bui M.M., Thomas L.B., Wilson C.P., DeLand L.A., Mastorides S.M. Lung and Colon Cancer Histopathological Image Dataset (LC25000). https://www.kaggle.com/datasets/andrewmvd/lung-and-colon-cancer-histopathological-images?resource=download.

